# Supplementary material for: The Impact of Quantifying Human Locomotor Activity on Examining Sleep–Wake Cycles
Source: Sensors (Basel). 2025 Dec 17;25(24):7659. doi: 10.3390/s25247659 (PMC12737032; doi:10.3390/s25247659)
Supplement: Supplementary file 1 [file sensors-25-07659-s001.zip › sensors-4012227-supplementary.pdf]

Supplementary Material for Manuscript Titled

The Impact of Quantifying Human Locomotor Activity on Examining Sleep–Wake Cycles

Bálint Maczák *, Adél Zita Hordós and Gergely Vadai

Department of Technical Informatics, University of Szeged, 6720, Szeged, Hungary; adelhordos@gmail.com (A.Z.H.); vadaig@inf.u-szeged.hu (G.V.)
* Correspondence: maczak@inf.u-szeged.hu

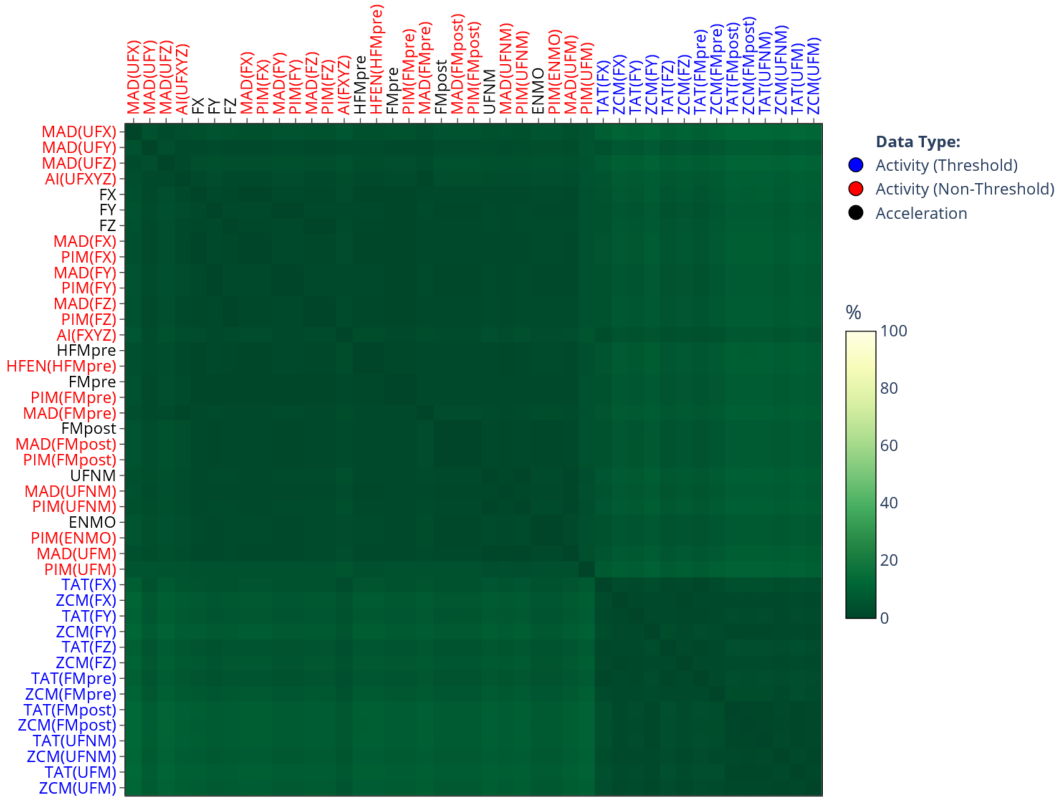

Figure S1. Differences in the mean value of M10 across 42 subjects depending on which signal-processing pipeline of our generalized framework was used to generate the acceleration or activity data. The cells of the matrix represent SMAPE values ranging from 0% to 200%, with the gradient color scale shifting linearly from green to yellow as SMAPE increases from 0% to 100%. The label colors follow the same color coding as in Figure 3, as indicated in the legend.

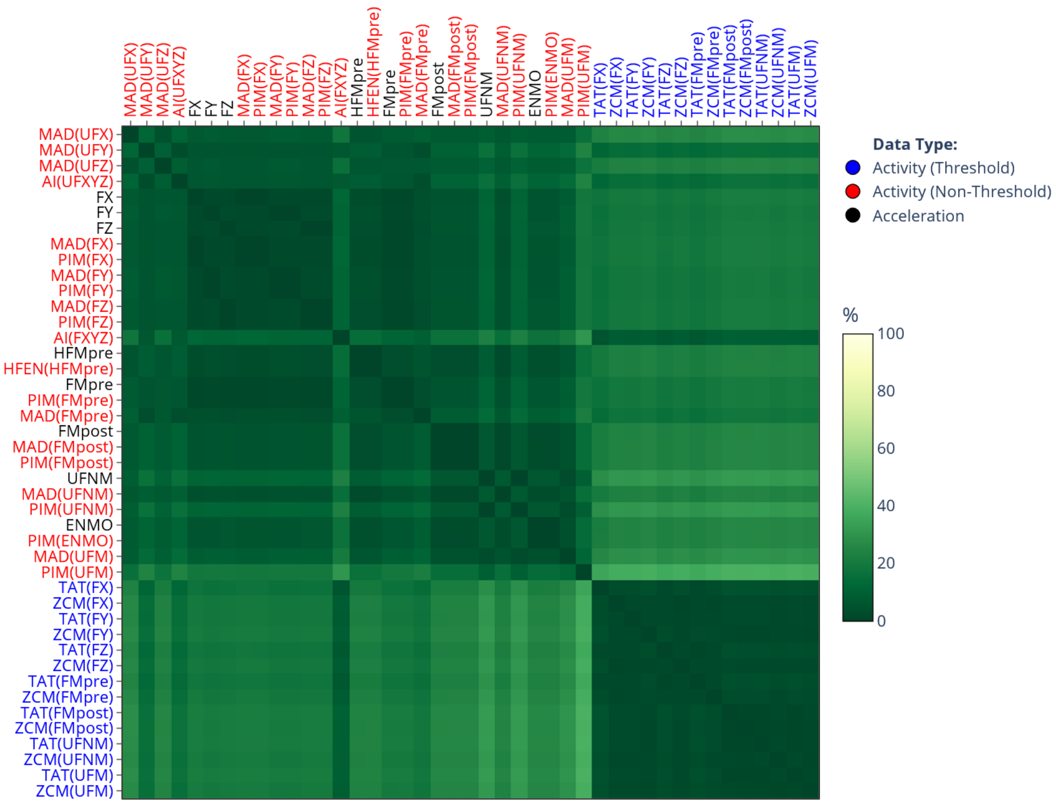

Figure S2. Differences in the mean value of RA across 42 subjects depending on which signal-processing pipeline of our generalized framework was used to generate the acceleration or activity data. The cells of the matrix represent SMAPE values ranging from 0% to 200%, with the gradient color scale shifting linearly from green to yellow as SMAPE increases from 0% to 100%. The label colors follow the same color coding as in Figure 3, as indicated in the legend.

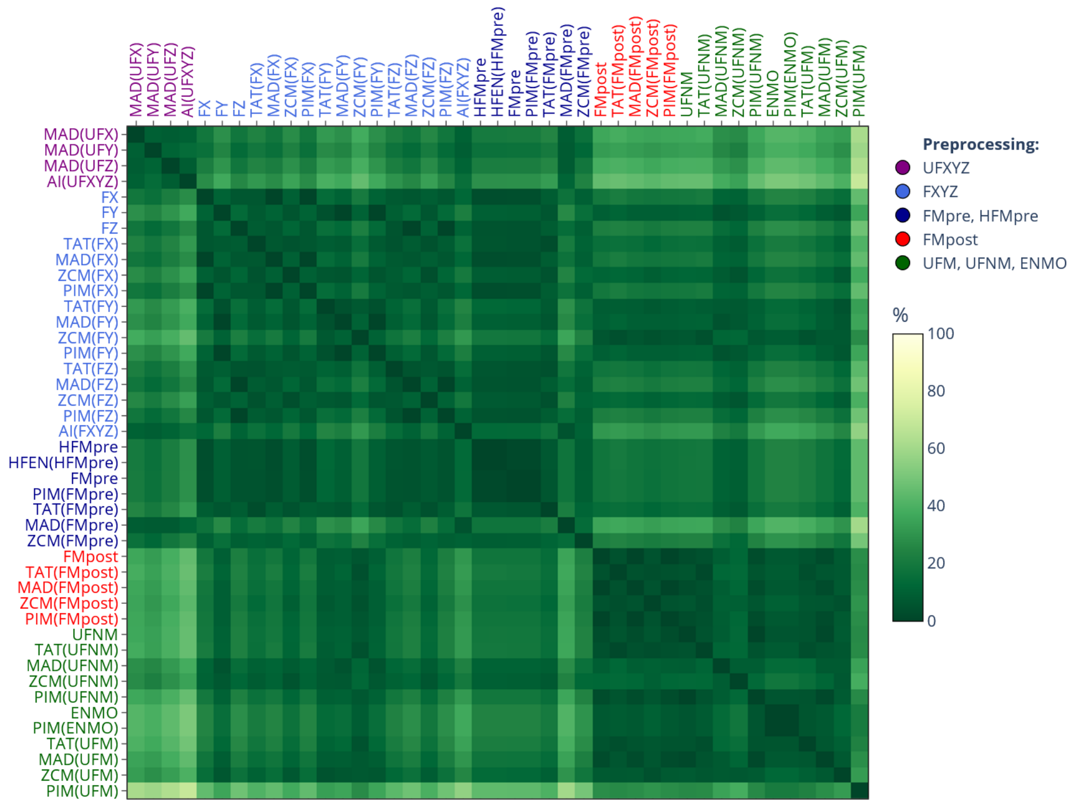

Figure S3. Differences in the mean value of IV across 42 subjects depending on which signal-processing pipeline of our generalized framework was used to generate the acceleration or activity data. The cells of the matrix represent SMAPE values ranging from 0% to 200%, with the gradient color scale shifting linearly from green to yellow as SMAPE increases from 0% to 100%. The label colors follow the same color coding as in Figures 2 and 4, as indicated in the legend.
